# Synthesis, crystal structure, Hirshfeld surface investigation and comparative DFT studies of ethyl 2-[2-(2-nitrobenzylidene)hydrazinyl]thiazole-4-carboxylate

**DOI:** 10.1186/s13065-022-00805-1

**Published:** 2022-03-22

**Authors:** Muhammad Haroon, Tashfeen Akhtar, Muhammad Yousuf, Muhammad Nawaz Tahir, Lubna Rasheed, Syeda Saniya Zahra, Ihsan ul Haq, Muhammad Ashfaq

**Affiliations:** 1 Department of Chemistry, Mirpur University of Science and Technology (MUST), 10250-Mirpur (AJK), Pakistan; 2 Department of Chemistry, Government Major Muhammad Afzal Khan (Shaheed), Boys Degree College Afzalpur, Mirpur, (Affiliated with Mirpur University of Science and Technology (MUST), 10250-Mirpur (AJK), Pakistan; 3 Department of Chemistry, Ulsan National Institute of Science and Technology (UNIST), Ulsan, South Korea; 4 Department of Physics, University of Sargodha, Sargodha, Punjab, Pakistan; 5 Department of Chemistry, Division of Science and Technology, University of Education, Township, Lahore, Pakistan; 6 Department of Pharmacy, Quaid-I-Azam University, 45320 Islamabad, Pakistan; 7 Department of Physics, University of Mianwali, Mianwali, Punjab, Pakistan

**Keywords:** Thiazole, DFT calculations, Anti-oxidant, XRD, Spectroscopy

## Abstract

The ethyl 2-[2-(2-nitrobenzylidene)hydrazinyl]thiazole-4-carboxylate (**1**), a thiazole ester, was synthesized by refluxing 1-(2-nitrobenzylidene)thiosemicarbazide and ethyl bromopyruvate. The compound is characterized by spectrometric, spectroscopic and single crystal (SC-XRD) techniques. Non-covalent interactions that are responsible for crystal packing are explored by Hirshfeld surface analysis. All theoretical calculations were performed by DFT quantum chemical methods using 6-311G(d,p) and cc-pVTZ basis sets and compared. Theoretical harmonic frequencies of ethyl 2-[2-(2-nitrobenzylidene)hydrazinyl]thiazole-4-carboxylate (**1**) were optimized. Confirmation of hydrogen bonding sites was analyzed by molecular electrostatic potential (MEP) and Mulliken population analysis. The vibrational frequencies of characteristic functional groups and chemical shifts were found in good agreement with experimental assignments. Frontier molecular orbital (FMO) revealed relatively small HOMO–LUMO (highest occupied molecular orbital-lowest unoccupied molecular orbital) gape, which speaks off the nearly planar geometry and extended conjugation, as compared to the substituents with no conjugation possible. It has also been observed that –NO_2_ substituent plays a vital role for this relatively small HOMO–LUMO gape and overall electronic properties when compared with similar thiazole carboxylates (**2–6**, Table 6). Ethyl 2-[2-(2-nitrobenzylidene)hydrazinyl]thiazole-4-carboxylate **(1)** was also evaluated for its anti-oxidant and anti-microbial activities.

**Graphical Abstract:**

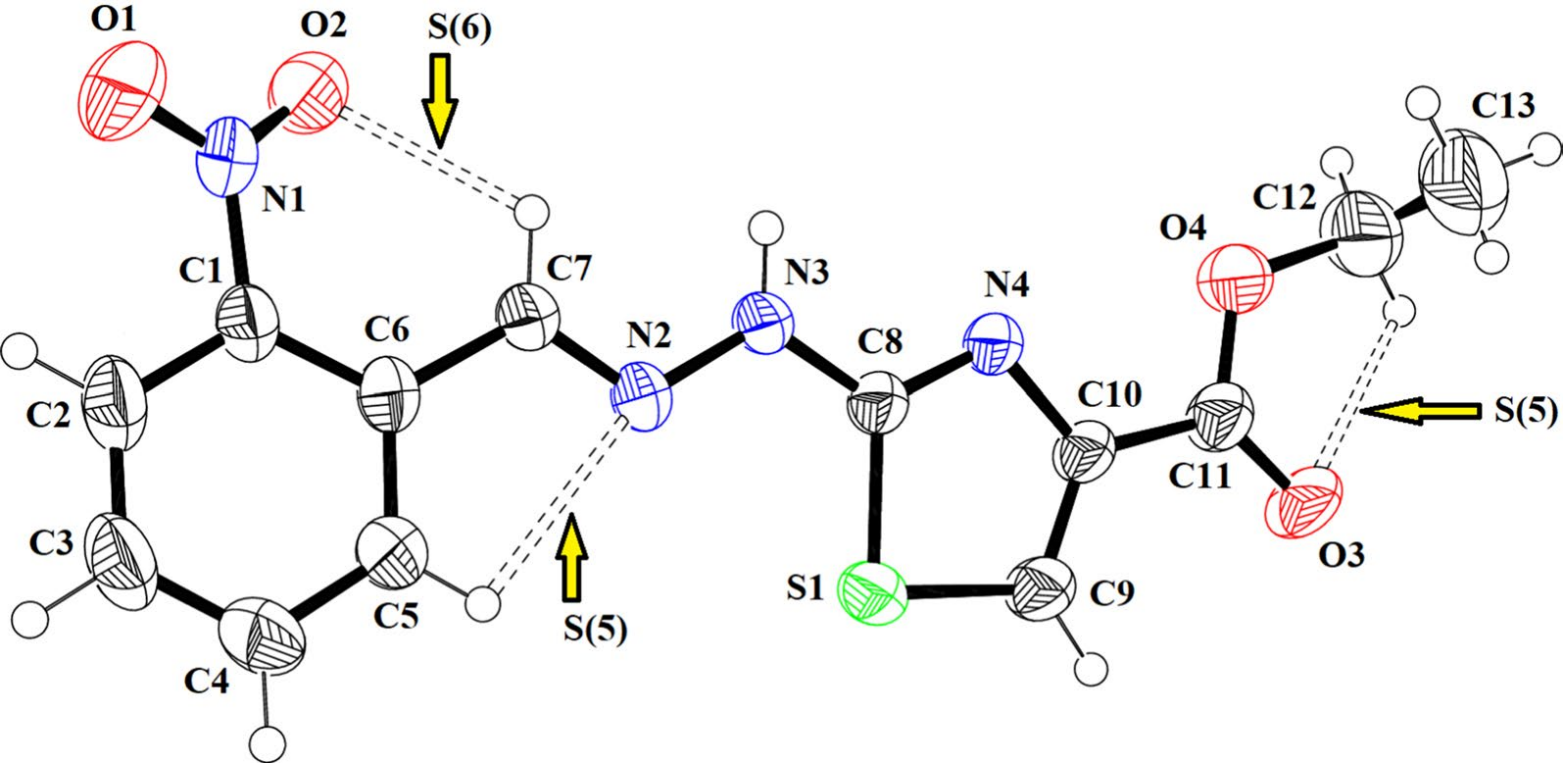

**Supplementary Information:**

The online version contains supplementary material available at 10.1186/s13065-022-00805-1.

## Introduction

1,3-Thiazole is an important sulphur and nitrogen containing azole [1]. Binding capability of sulphur and nitrogen in 1,3-thiazole, to induce sigma and pi interactions with receptor moieties, makes it an interesting class of heterocycles in drug designing [2]. It is very significant motif in pharmaceutical chemistry [3–5]. Thiazole derivatives are known for their diverse biological significance like anticancer [6], antiallergic [7], antiviral [8], antibacterial [9], antifungal [10], antitubercular [11, 12] and antiinflammatory [13–15] activities. Besides these, antiparasitic agents viz*,* nitazoxanide, tenonitrozole and aminitrozole have 1,3-thiazole ring in their structure [16].

Computational chemists use both experimental data and theoretical models in order to determine the structural features like bond lengths, bond angles and torsion angles besides accounting for spectroscopic properties like harmonic frequencies, vibrations and chemical shifts at molecular level. An important tool in the hand of theoretical chemists to compare and analyze the properties like geometry, vibrational frequency and dipole moment [17–24] of the molecules, is Density Functional Theory (DFT). The thiazole ester, ethyl 2-[2-(2-nitrobenzylidene)hydrazinyl]thiazole-4-carboxylate (**1**) was synthesized for the purpose of biological studies. However, it was found interesting to investigate the structural features by comparing theoretical and experimental data and also to see the effect of the substituent i.e. nitro group (–NO_2_), on the conjugation leading to difference in the HOMO–LUMO gape with extended conjugation. The analysis of theoretical results revealed that –NO_2_ substituent has played a vital role in decreasing the HOMO–LUMO gape, furthermore; it has also exerted its effect on overall electronic properties when compared with compounds **2–6**.

## Materials and methods

**Scheme 1:**
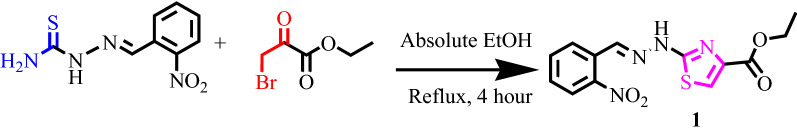
Synthesis of ethyl 2-[2-(2-nitrobenzylidene)hydrazinyl]thiazole-4-carboxylate

Ethyl 2-[2-(2-nitrobenzylidene)hydrazinyl]thiazole-4-carboxylate (1) was synthesized by the reaction of 1-(2-nitrobenzylidene)thiosemicarbazide and ethyl bromopyruvate (Scheme 1). The pure reagent grade chemicals were purchased and used as such. Thiosemicarbazide, 4-nitrobenzaldehyde and ethyl bromopyruvate were purchased form Merck Germany. Pre-coated Silica 60 HF_254_ Aluminum sheets (Merck, Germany) were used to monitor the reaction with thin layer chromatography (TLC). The melting point determination, functional group identification, NMR chemicals shifts and single crystal analysis were carried out as reported elsewhere [25]. Dimethyl sulfoxide-deuterated (DMSO-*d*_*6*_) was used to record the proton and carbon NMR (nuclear magnetic resonance), using 300 and 75 MHz frequency, respectively. High resolution mass spectrometry (HRMS) was carried out on Bruker Micro TOF-ESI (time of flight-electrospray ionization) spectrometer, positive targeted mode.

### Synthesis of ethyl 2-[2-(2-nitrobenzylidene)hydrazinyl]thiazole-4-carboxylate (1)

The desired compound (**1**) was obtained by refluxing 1-(2-nitrobenzylidene)thiosemicarbazide (0.5 g, 0.00237 mol) and ethyl bromopyruvate (0.3 mL, 0.00238 mol) for 4 h in absolute ethanol (20 mL). The reaction progress assessed by TLC. When the reactants consumed, as shown by TLC, quenching of reaction by adding ice cold water resulted into precipitates, which on filtration and washing with excess of water yielded pure compound [25].

Colour: Yellow solid; Yield: 82%; Melting point: Above 300 °C; R_f_: 0.52 (acetone/*n*-hexane, 1:2); **FTIR** (ATR, cm^−1^): ν̅ 1076, 1338, 1436, 1517, 1571, 1684, 2981; ^**1**^**H–NMR**: δ (ppm) 1.28 (3H, t, –CH_3_, *J* = 7.2 Hz), 4.24 (2H, q, –OCH_2_–CH_3_, *J* = 7.0 Hz), 7.60 (1H, dt, Ar–H, *J* = 1.5 Hz, *J* = 8.4 Hz), 7.78 (1H, t, Ar–H, *J* = 7.7 Hz), 8.02 (2H, m, Ar–H), 7.82 (1H, s, 1,3-thiazole ring C-5), 8.37 (1H, s, –CH=N–), 12.63 (1H, s, –N–NH–C–); ^**13**^**C–NMR**: δ (ppm) 14.6 (–CH_3_), 60.9 (–OCH_2_–), 120.2 (C-5, 1,3-thiazole ring), 125.2, 128.1, 128.7, 130.4, 137.4, 147.9 (Ar–C), 137.6 (–CH=N–), 143.3 (C-4, 1,3-thiazole ring), 161.7 (C=O), 168.2 (C-2, 1,3-thiazole ring); **HRMS**: 321.9680 **[M + H]**^**+**^, 343.0700 **[M + Na]**^+^.

### Single Crystal X-Rays Diffraction (SC-XRD) studies

The crystallization of ethyl 2-[2-(2-nitrobenzylidene)hydrazinyl]thiazole-4-carboxylate (**1**) from tetrahydrofuran (THF) yielded the crystals suitable for diffraction collection using single crystal X-ray diffractions. Table 1 presents the experimental conditions, different parameters and structure refinements. The data reduction and cell refinement carried out using SAINT and data collection by APEX 2 [26]. SHELXS97 [27] and SHELXL97 [28] were used for solving and refining the structure, respectively. PLATON [29], Mercury 3.6 software and ORTEP-3 for Windows [30] were used to draw molecular graphics. The CIF is deposited at Cambridge Crystallographic Data Centre (CCDC = 1,951,603).

**Table 1:** SC-XRD experimental details of ethyl 2-[2-(2-nitrobenzylidene)hydrazinyl]thiazole-4-carboxylate (**1**)

Crystal data	
*Chemical formula*	C_13_H_12_N_4_O_4_S
*M*_r_	320.33
Crystal system, space group	Monoclinic, *P*2_1_/*c*
Temperature (K)	296
*a*, *b*, *c* (Å)	14.423 (3), 11.270 (2), 9.2375 (16)
β (°)	105.510 (7)
*V* (Å^3^)	1446.9 (5)
*Z*	4
Radiations	Mo *K*α
µ (mm^−1^)	0.25
Crystal size (mm)	0.38 × 0.18 × 0.16
*Data collection*	
Diffractometer	Bruker Kappa APEXII CCD
Absorption correction	Multi-scan (*SADABS*; Bruker, 2005)
*T*_min_, *T*_max_	0.895, 0.970
No. of measured, independent and observed [*I* > 2σ(*I*)] reflections	9460, 3409, 1921
*R*_int_	0.060
(sin θ/λ)_max_ (Å^−1^)	0.658
*Refinement*	
*R*[*F*^2^ > 2σ(*F*^2^)], *wR*(*F*^2^), *S*	0.061, 0.166, 1.01
No. of reflections	3409
No. of parameters	200
H-atom treatment	H-atom parameters constrained
Δρ_max_, Δρ_min_ (e Å^−3^)	0.36, − 0.29

## Results and discussion

The synthesis of ethyl 2-[2-(2-nitrobenzylidene)hydrazinyl]thiazole-4-carboxylate (1) was achieved in two steps form thiosemicarbazide via thiosemicarbazone and cyclization with ethyl bromopyruvate. The synthesis was confirmed by spectroanalytical techniques. The spectroscopic data verified the proposed synthesis and the structure was later confirmed by the SC-XRD.

### Single crystal X-ray

**Fig. 1:**
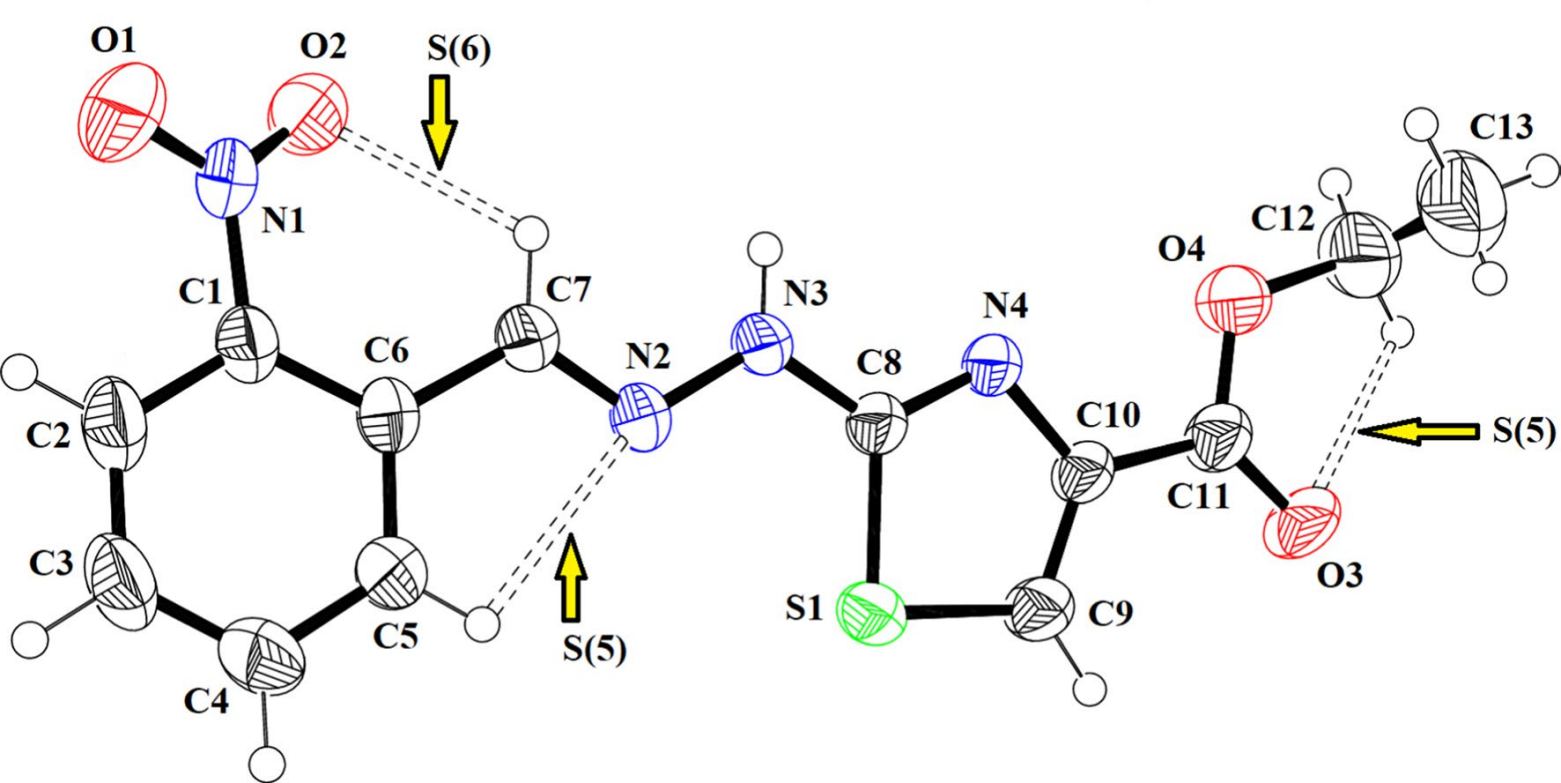
The ORTEP diagram drawn at probability level of 50% for **(1)**. H-atoms are displayed by tiny circles of arbitrary radii

**Fig. 2:**
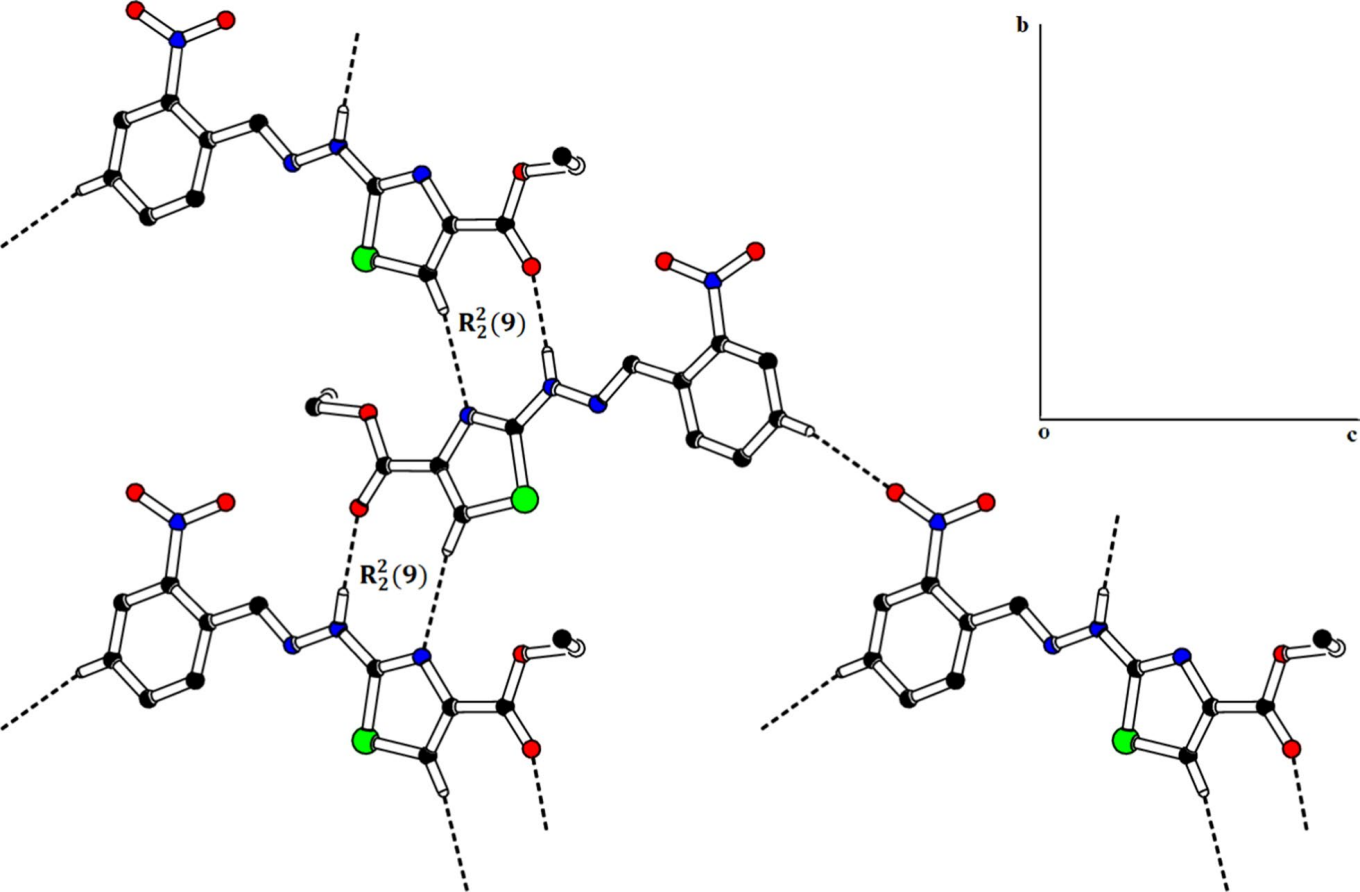
Packing diagram showing 2D sheet formed by N–H…O, C–H…N and C–H…O bonding for **(1)**

**Fig. 3:**
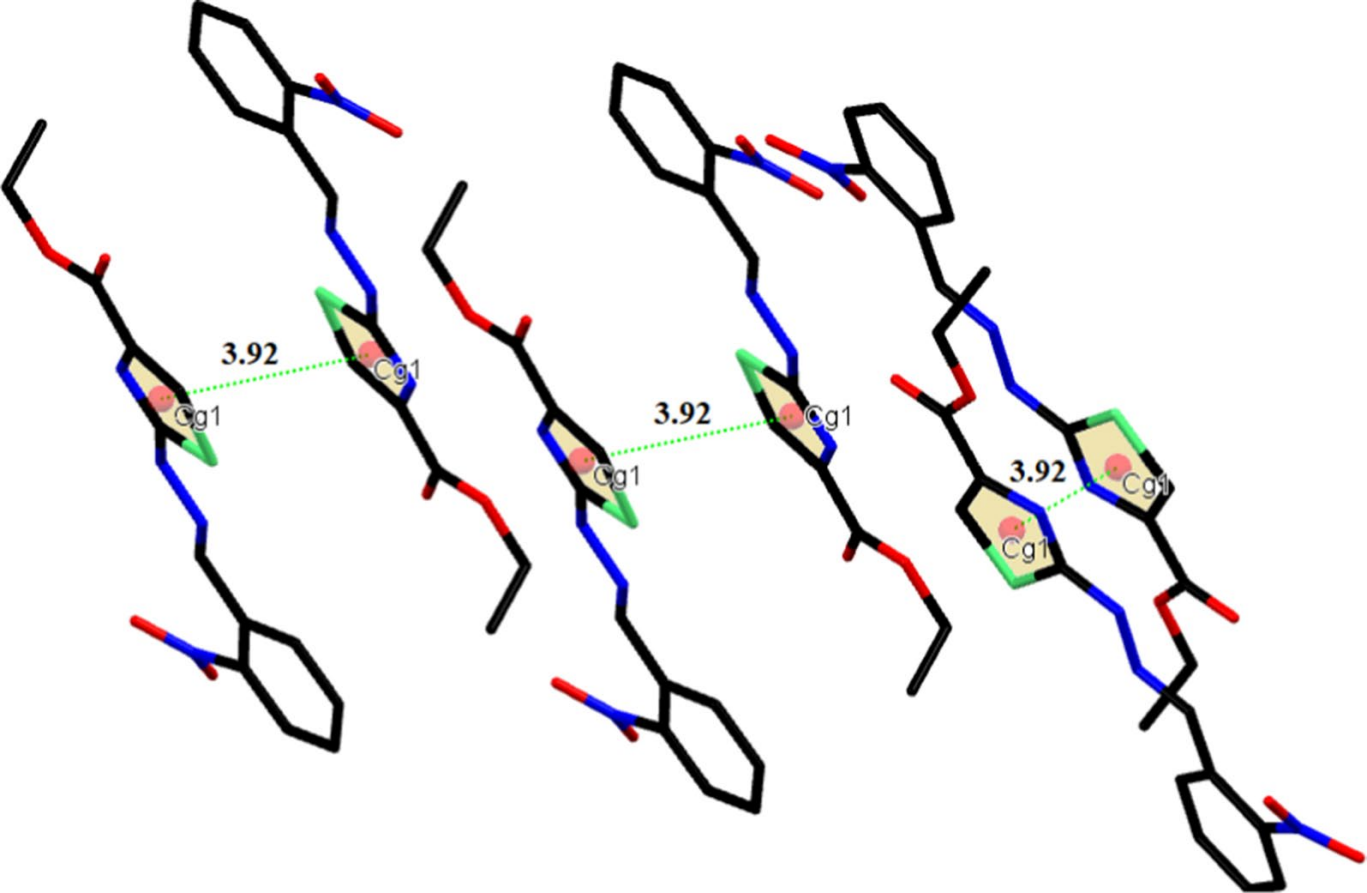
Graphical view of π–π stacking interaction for **(1)**. Distance shown is measured in Å. H-atoms are omitted for clarity

The SC-XRD inspection showed that ethyl 2-[2-(2-nitrobenzylidene)hydrazinyl]thiazole-4-carboxylate (**1) (**Fig. 1) exists in *E*-configuration, the benzylidenehydrazinyl moiety *A* (C1–C7/N1/N2) and the thiazole ring *B* (C8/N4/C9/C10/S1) are found to be planar with respective root mean square (r. m. s.) deviations of 0.0287 and 0.0032 Å. The dihedral angle between A/B is 8.41 (9)°. The planarity of the thiazole ring in two most closely related reported crystal structures, one having methyl substituted phenyl ring and second having bromo substituted phenyl ring was obvious by r.m.s. deviation of 0.004 and 0.0072 Å [25]. The nitro group C (N1/O1/O2) and carboxylate group D (C11/O3/O4) are oriented at a dihedral angle of 26.84 (10)° and 4.6 (5)° with the parent groups A and B, respectively. The C-atoms (C12/C13) are at a distance of -0.003 (11) and 1.329 (13) Å, respectively, from the carboxylate group as compared to their distance of 0.034 (16) and 1.440 (21) Å from the carboxylate group in closely related crystal structure having bromo substituted phenyl ring instead of nitro substituted phenyl ring [25]. Intramolecular H-bonding of type C–H…N and C–H…O are responsible for stabilization of molecular configuration. CH of imine functional group is found to engage with O-atom of nitro group (O2) to form S(6) loop through intra C–H…O bonding. Similarly, N-atom of imine functional group is found to engage with CH of phenyl ring to form S(5) loop through C–H…N bonding. Another S(5) is formed by C–H…O bonding, where O-atom is from carboxylate group and CH is from alkyl group (C12/C13). These intramolecular H-bonded loops are displayed in Fig. 1 and specified in Table 2. The molecules are primarily connected to each other in the form of dimers through strong N–H…O and comparatively weak C–H…N bonding to form ${\mathrm{R}}_{2}^{2}(9)$ loop as displayed in Fig. 2 and specified in Table 2 [32]. Infinite C7 chain is formed by N3-H3A…O3 bonding whereas C4 chain is formed by C–H…N bonding. These chains extend along b crystallographic axis in a zigzag fashion. The molecules are also interlinked by C–H…O bonding, where *para* CH of phenyl ring (C1–C6) acts as donor and O-atom is from nitro group. Due to this C–H…O bonding, another zigzag C6 chain is formed along c crystallographic axis. Besides the role of H-bonding in packing of molecules, weak non-covalent interaction named as off-set π–π stacking interaction also play significant role in the crystal packing. Five membered ring of one molecule located at asymmetric location is engaged in off-set π–π stacking interaction with the five membered ring of neighboring symmetry related molecule (-x,1-y,-z). Centroid to centroid distance and ring off-set for this particular interaction are found to be 3.92 Å and 1.798 Å, respectively as displayed in Fig. 3.

**Table 2:** Hydrogen-bond geometry (Å, º) for (1)

*D*—H···*A*	*D*—H	H···*A*	*D*···*A*	*D*—H···*A*
N3—H3*A*···O3^i^	0.86	2.03	2.860 (3)	161
C5—H5···N2	0.93	2.44	2.764 (4)	101
C7—H7···O2	0.93	2.28	2.812 (4)	116
C12—H12A···O3	0.97	2.30	2.679 (5)	102
C9—H9···N4^ii^	0.93	2.58	3.502 (4)	171
C3—H3···O1^iii^	0.93	2.55	3.432 (4)	159

Symmetry codes: (i) − *x*, *y* − 1/2, − *z* − 1/2; (ii) − *x*, *y* + 1/2, − *z* − 1/2; (iii) − *x* + 1, *y* + 1/2, − *z* + 3/2

### Hirshfeld surface analysis (HSA)

**Fig. 4:**
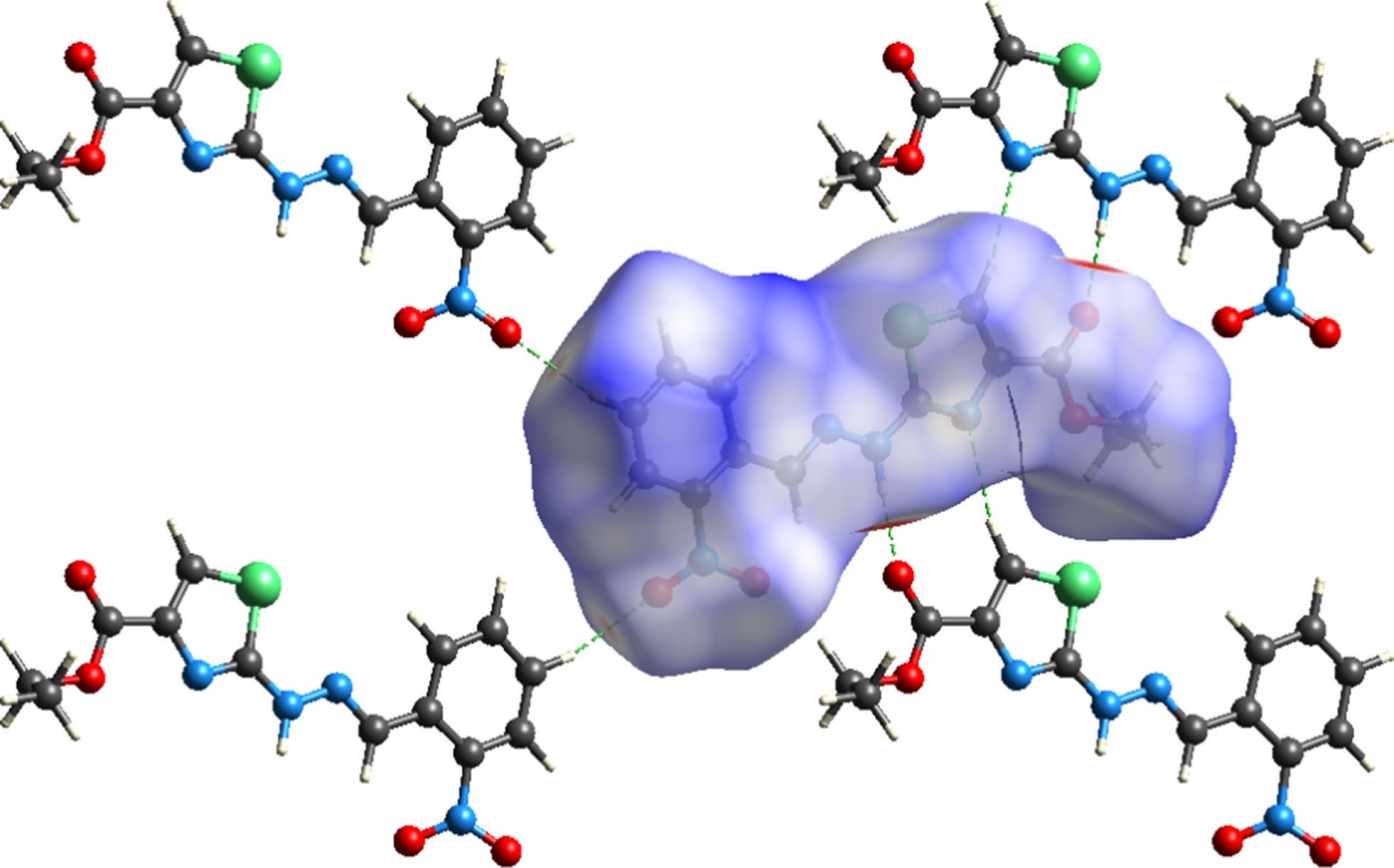
HS plotted over d_norm_ in the range − 0.5540 to 1.4207 a.u. for **(1)**

**Fig. 5:**
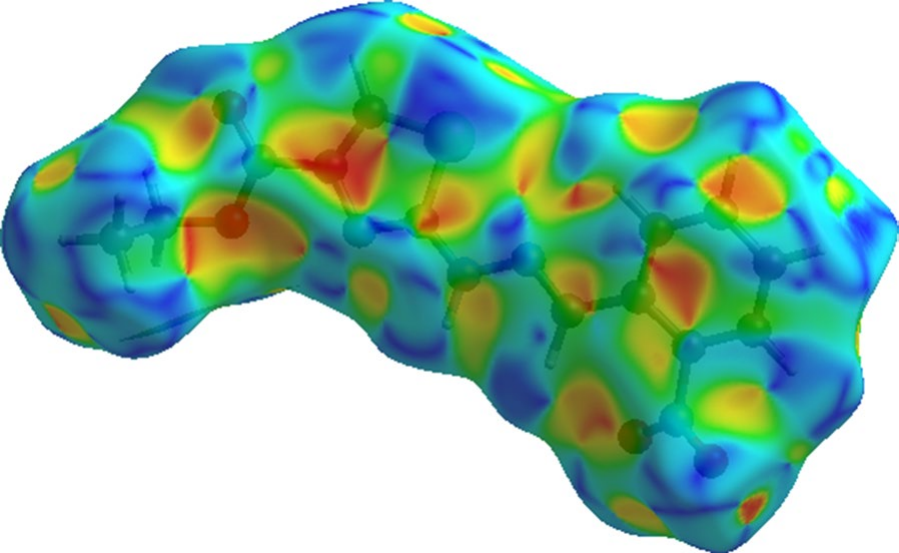
HS plotted over shape index in the range − 1 to 1a.u. for **(1)**

**Fig. 6:**
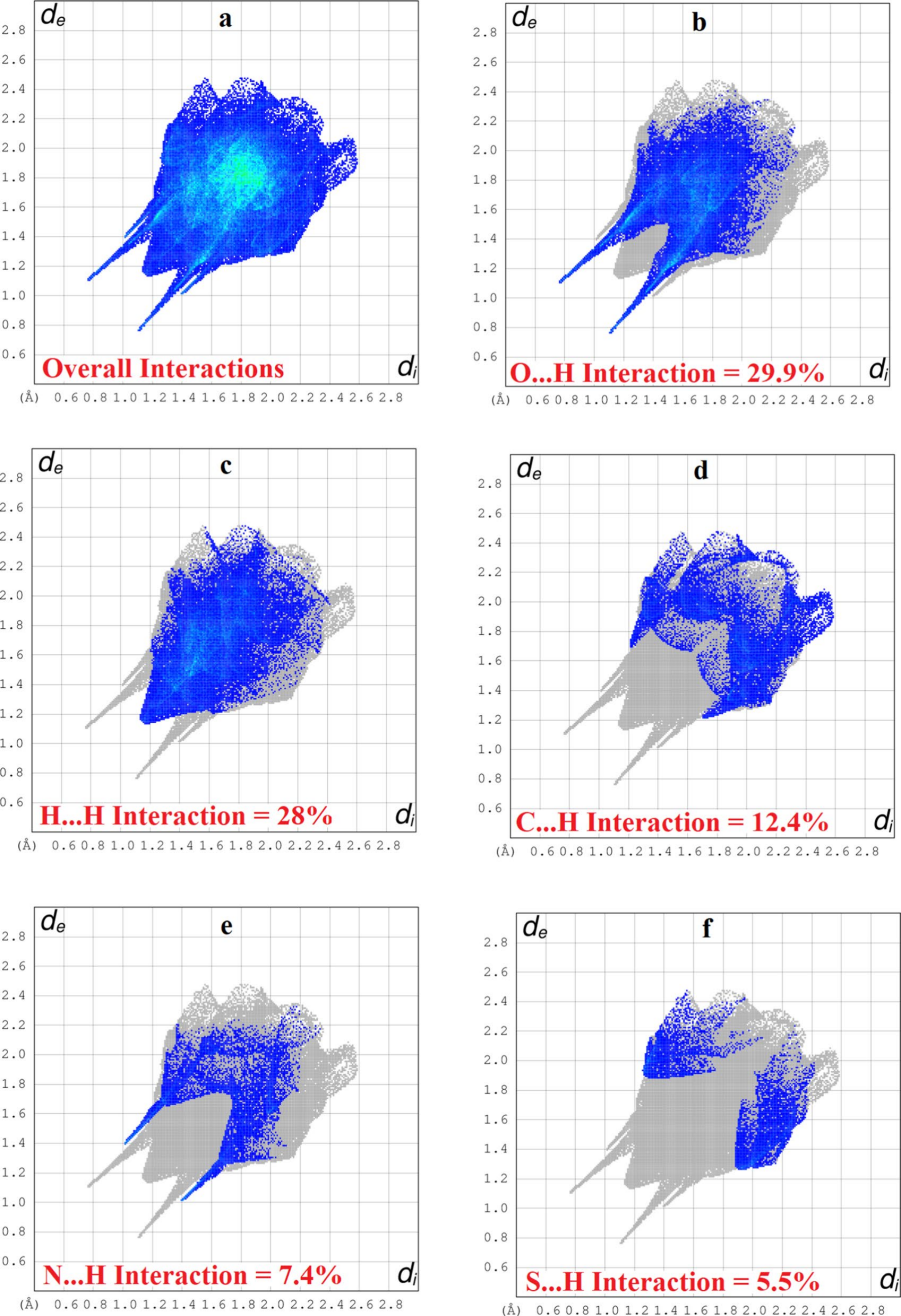
2D plots for **(1)**, **a** for overall interaction, **b**–**f** for major contributor in crystal packing

**Fig. 7:**
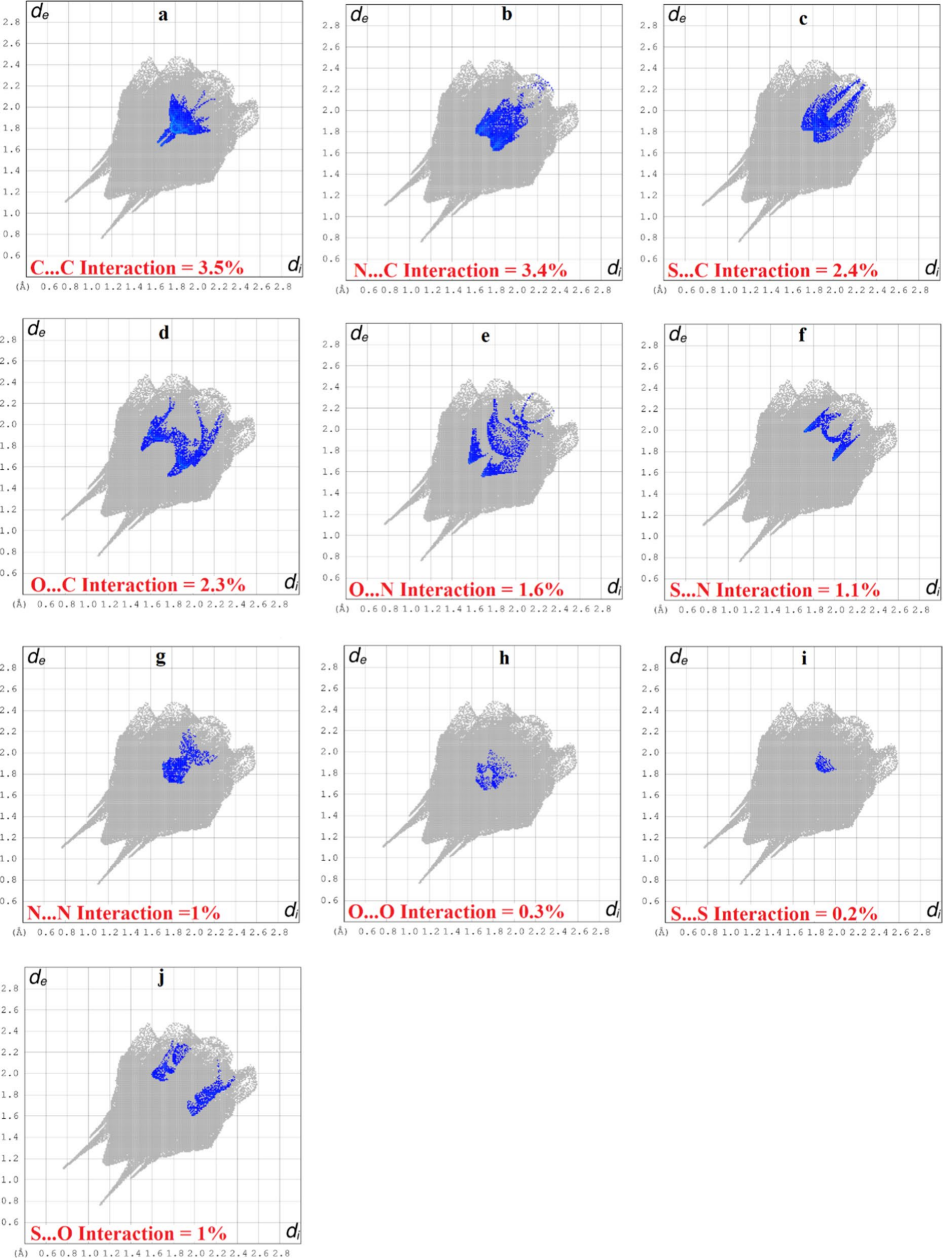
**a–j **2D plots for minor contributor in crystal packing for **(1)**

**Fig. 8:**
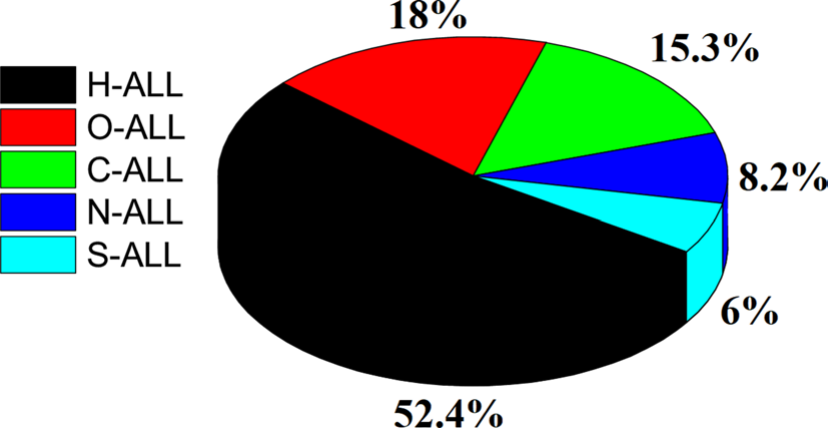
Percentage contribution of interaction of an atom located inside HS to atoms of molecules located in the surrounding of HS

For further exploration of non-covalent interactions that are accountable for the packing of molecules, Hirshfeld surface inspection is conducted on Crystal Explorer 17.5 [33]. Hirshfeld surface (HS) plotted over d_norm_ can be utilized to represent intermolecular interactions by colour coding [34–36]. The atomic contacts having distance shorter, equal and longer than summation of Van der Waals radii are represented by red, white and blue regions on HS, respectively. Bright red spots on the HS near NH group, carbonyl O-atom, *para* CH of nitro phenyl ring, one of the O-atom of nitro group indicate that these atoms are involved in H-bonding as displayed in Fig. 5. N-atom and CH of five membered ring are also engaged in H-bonding but no red spot is obvious in Fig. 4 due to graphical limitations. No red spot is found on the HS near imine N-atom indicating that this atom is not engaged in any kind of H-bonding. HS plotted over shape index is utilized to check that whether π–π stacking interaction is present in crystal packing or not. Consecutive triangular regions of red and blue on the HS around the five membered ring indicated that π–π stacking interaction is present in crystal packing as displayed in Fig. 5. As these triangular regions on the HS are not exactly in the center of five membered ring suggesting off-set π–π stacking interaction. In order to find out the percentage contribution of each interatomic contact involved in crystal packing, 2D fingerprint plots are utilized. In these plots, d_i_ and d_e_ stands for distance from the HS to nearest atom inside and outside the HS, respectively. 2D fingerprint plot for overall interactions in the crystal packing is displayed in Fig. 6a. The sky blue region in the center of this plot indicates the existence of π–π stacking interaction in crystal packing. The contribution of each individual interatomic contact is calculated including reciprocal contact. It is found that O…H, H…H, C…H, N…H and S…H interatomic contacts are the major contributors in crystal packing with percentage contribution of 29.9%, 28%, 12.4%, 7.4% and 5.5%, respectively as displayed in Fig. 6b-f. C…C, N…C, S…C, O…C, O…N, S…N, N…N, O…O, S…S, and S…O interatomic contacts are found to be minor contributor in crystal packing with percentage contribution of 3.5%, 3.4%, 2.4%, 2.3%, 1.6%, 1.1%, 1%, 0.3%, 0.2% and 0.1%, respectively as displayed in Fig. 7a–j. Furthermore, the interaction of an atom located inside the HS is computed with the atoms of molecules present in the surrounding of HS to have an idea which atom of a molecule is interacting strongly with the atoms of neighboring molecules. H-atoms located inside the HS interact strongly with the atoms of molecules in the neighbouring of HS, percentage contribution of this interaction is found to be 52.4%. The percent (%) contribution of all such interactions are displayed in Fig. 8.

### Quantum chemical calculations

**Fig. 9:**
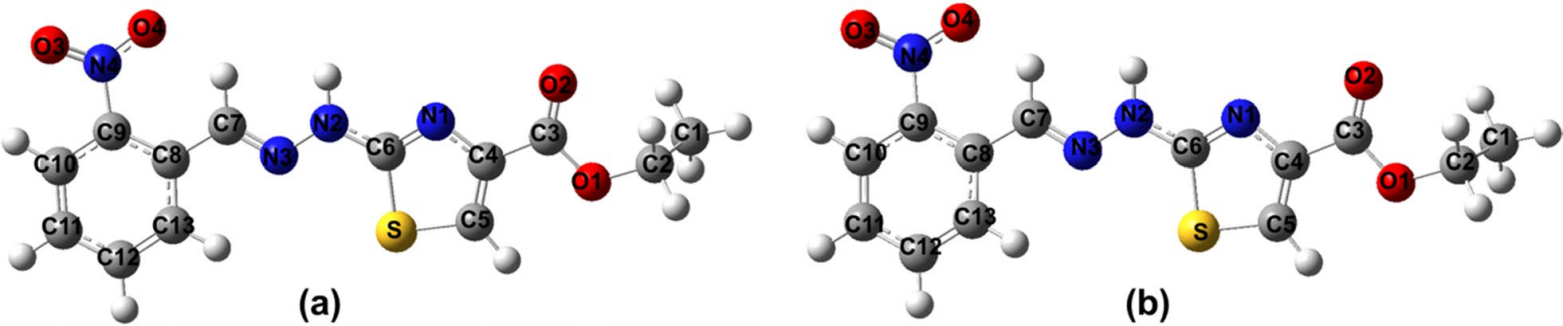
Optimized structure of compound (**a**) 6-311G(d,p) (**b**) ccPVTZ

**Fig. 10:**
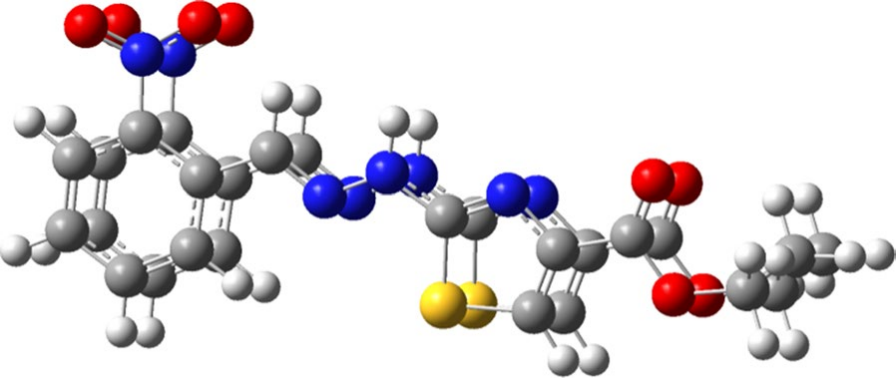
Overlay structure of compound optimized by 6-311G (d,p) and cc-pVTZ method

Guassian09 package [37–39] and Gauss-View molecular visualization software [40] was used in order to carry out calculation studies on personal computer. DFT (DFT/B3LYP) method [41, 42] with 6-311G(d,p) and cc-pVTZ basis sets were used for geometry optimization and further calculations (Fig. 9). The optimized structures obtained from these two methods were compared (Fig. 10) which showed almost same results. Optimized geometry was used in order to ascertain different aspects like vibrational spectra, chemical shifts, quantum chemical and thermodynamic properties of the compound, theoretically [43].

### Vibrational spectra

The vibrational spectra of the compound were computed theoretically (DFT/B3LYP method with 6-311G(d,p) and cc-pVTZ basis sets). The results obtained by two methods were subjected to scaling factors of 0.966 and 0.966 [44, 45] for 6-311G(d,p) and cc-pVTZ basis sets, respectively. The vibrational modes of the compound are assigned by animation of Guass View software, for both used methods. Total energy distribution (TED) calculations for compound (**1**) were executed with the scaled quantum mechanical (SQM) program [46, 47]. Several factors were taken into account for matching of the experimental and theoretical vibrational data of the compound (**1**), such as: animation option of the Gauss View package program, relative intensities of the vibrational bands and previously reported data in the literature [37]. The compound (**1**) contains 34 atoms and it shows 96 normal vibrational modes having a member of C point group with identity (E) symmetry operation. The conformer having the lowest optimization energy has been selected for calculating vibrational spectra of the molecule. At the end all the experimental results were compared to the theoretical results. Theoretical findings for infrared wavenumbers together with experimental results and TED values of the compound (**1**) are given in supporting information (Additional file 1: Table S1).

**Fig. 11:**
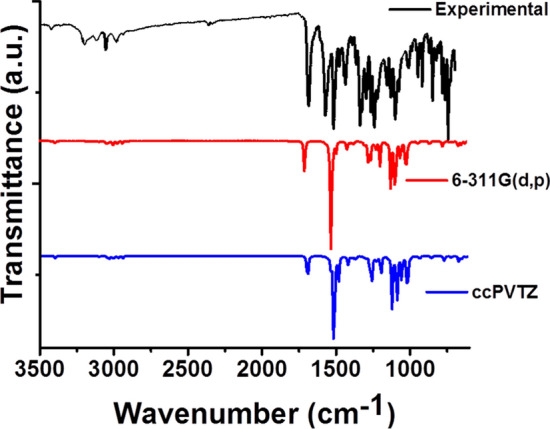
Comparison of FT-IR spectra with experiment and calculated values (DFT/B3LYP method with the 6-311G(d,p) and cc-pVTZ basis sets)

In the high wavenumber region bands appeared at around 3493 cm^−1^ are due to NH vibrations while those appeared at 3273–3036 cm^−1^ are due to symmetric C–H vibrations. The broadened shape of the band at this region could be attributed to the indication of residue of moisture in the sample and intra and intermolecular interactions. The band appeared at 1685 and 1571 cm^−1^ are due to C = O and –NO_2_ stretchings respectively [48]. The bands at 1298, 1251, 1244, 1160 and 1103 cm^−1^ are due to C–H stretching. The bands at 1336 and 742 cm^−1^ are due to C–C stretching [49, 50]. For the sake of better comparison experimental and calculated results were plotted as shown in Fig. 11.

### NMR spectra

The ^1^H- and ^13^C–NMR spectra of the compound (**1**) were obtained in deuterated dimethyl sulfoxide and signals calibrated with respect to TMS and assigned the chemical shifts to respective protons and carbons on the basis of the chemical environment. The proton NMR spectrum exhibited a triplet and quartet at 1.28 and 4.24 ppm corresponding to methyl and methylene of ethyl ester moiety in the molecules, respectively. The ortho substitution of the benzene ring was confirmed by the presence of a triplet and a triplet of doublets at 7.60 and 7.78 ppm, respectively. The triplet of doublets indicates one small coupling constant (*J* = 1.5 Hz) corresponding to *meta* splitting and one large coupling constant (*J* = 8.4 Hz) for the *ortho* splitting. Two remaining aromatic protons were multiplet at 8.04–7.99 ppm. The singlet at 7.82 ppm, integrating for one proton, was assigned to thiazole ring proton. The benzylidene –CH– proton was observed as singlet at 8.37 ppm. The –NH proton was observed as a downfield singlet at 12.63 ppm.

The carbon NMR spectrum showed thirteen signals corresponding to total number of carbons in the compound. Two signals in aliphatic region at 14.6 and 60.9 ppm were assigned to the ethoxy carbons i.e. –CH_3_ and –OCH_2_, respectively. The six aromatic carbons of benzene ring were seen between 125.2 to 147.9 ppm. The benzylidene –CH– and thiazole ring C-4 was observed at 137.6 and 143.3, respectively. The carbonyl of ester and thiazole C-2 were observed at 161.7 and 168.2 ppm, respectively. The proton and carbon spectra confirmed the proposed structure for the synthesized compound.

**Fig. 12:**
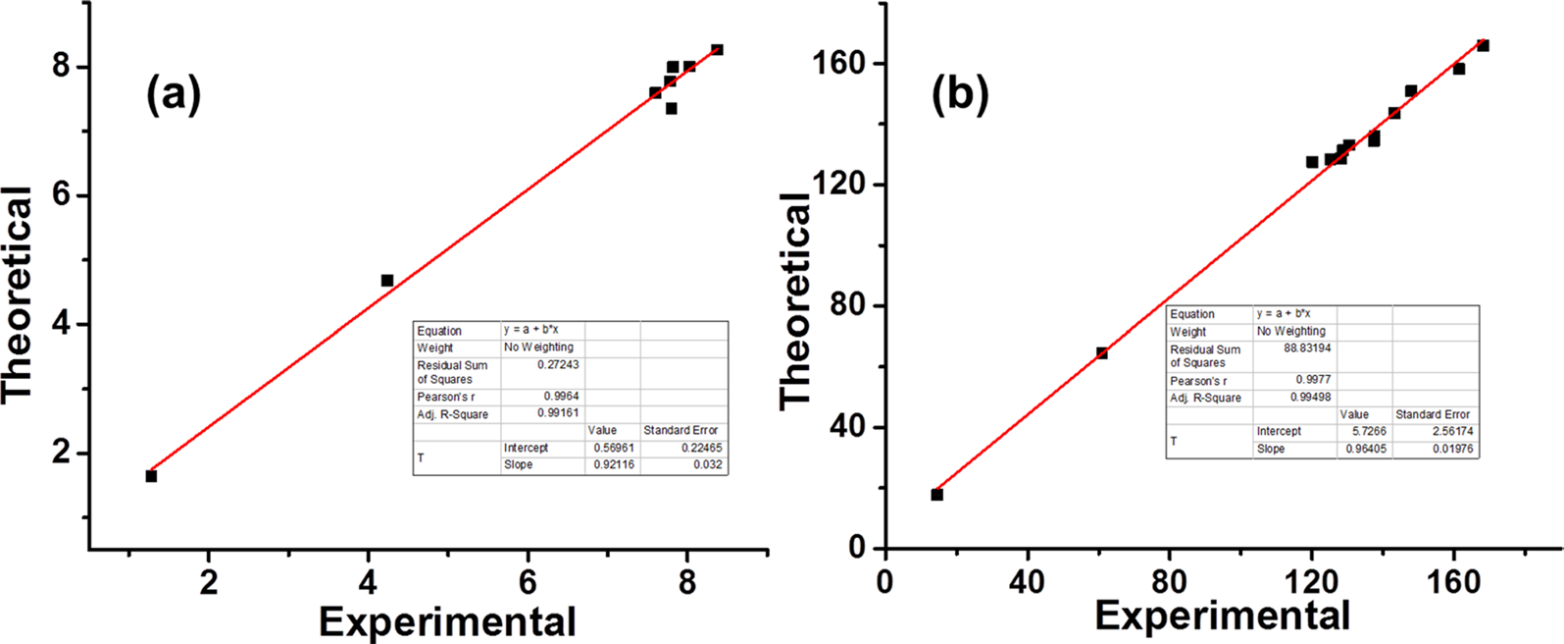
Correlation graphics of calculated and experimental chemical shifts of compound (**a**) ^1^H–NMR (**b**) ^13^C–NMR by B3LYP/6-311G(d,p)

**Fig. 13:**
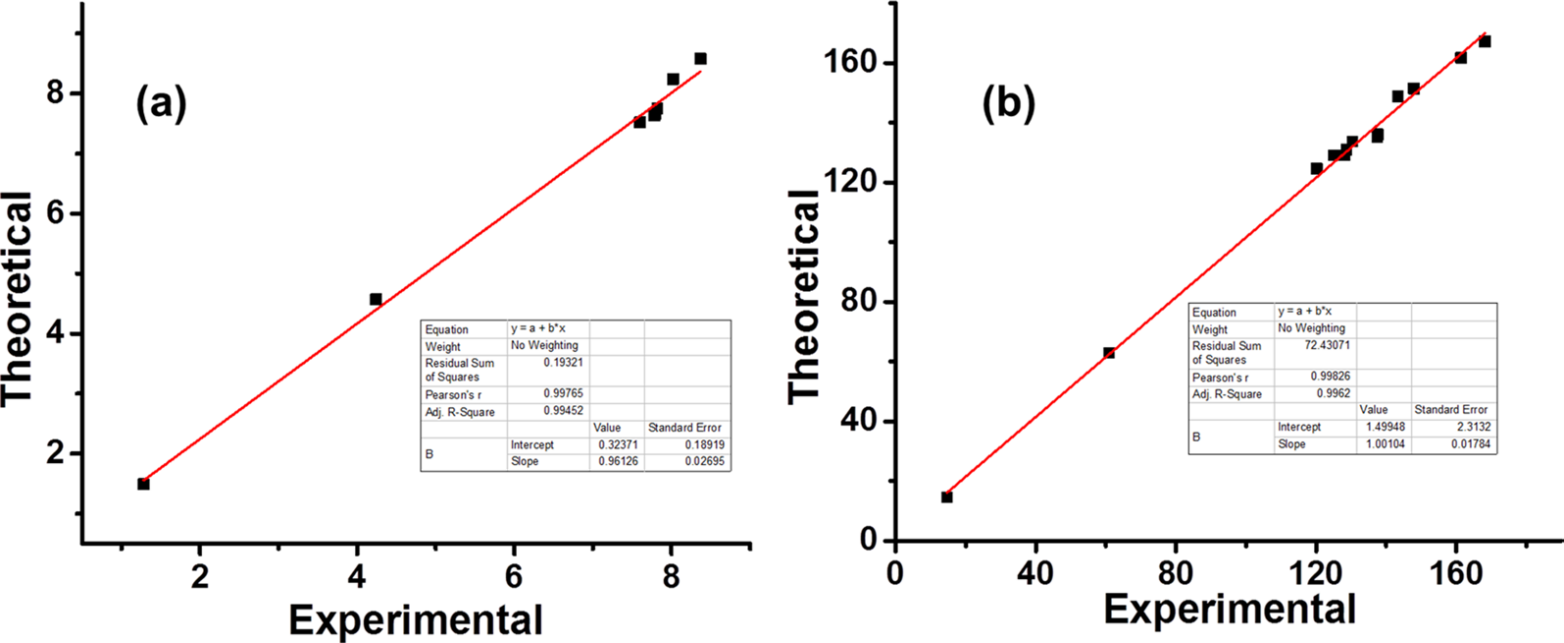
Correlation graphics of calculated and experimental chemical shifts of compound (**a**) ^1^H–NMR (**b**) ^13^C–NMR by B3LYP/cc-pVTZ

GIAO approach was used in order to calculate chemical shifts using B3LYP/6-311G(d,p) and B3LYP/cc-pVTZ, which are then converted to the TMS scale as reported earlier [25] using the formula i.e. δ = Σo–Σ. IEF-PCM (Integral-Equation-Formalism Polarizable Continuum Model) model was employed in order to incorporate the solvent effects [51]. Correlations graphs were produced for comparison of theoretically calculated NMR results to experimental values (Figs. 12 and 13). The correlation co-efficients for B3LYP/6-311G(d,p) (Fig. 12) are 0.9964 for –^1^H and 0.9977 for –^13^C NMR, while correlation co-efficients for B3LYP/cc-pVTZ (Fig. 7) 0.9976 for –^1^H and 0.9982 for –^13^C NMR were observed which reflects better agreement in experimental and theoretical results. The experimental and theoretically calculated NMR data by both methods has been presented in supporting information (Additional file 1: Tables S2, S3).

### HOMO and LUMO analysis

Frontier molecular orbitals (FMO) have unique importance for calculation of electronic states and stability of a molecule because these orbitals are at frontiers of electron occupation i.e. highest occupied molecular orbital (HOMO) and lowest lying unoccupied molecular orbital (LUMO) [44]. Theoretically calculated HOMO and LUMO are shown in Fig. 8. The compound possessed 83 occupied molecular orbitals. The respective energies for HOMO and LUMO are − 6.1093 and − 2.8798 eV for B3LYP/6-311G(d, p) levels and − 6.1148 and − 2.8763 for B3LYP/cc-pVTZ levels, respectively. Ionization potential (*I*), electron affinity (*A*) electronegativity (*χ*), chemical hardness (*η*) and chemical softness (*S*) have been obtained from FMO data (Table 3) [52]. Both methods produced almost the same results.

**Table 3:** The calculated FMO parameters using B3LYP/6-311G(d, p) level

Parameters	B3LYP/6-311G(d, p)	B3LYP/cc-pVTZ
*E*_*HOMO*_	− 6.1093	− 6.1148
*E*_*LUMO*_	− 2.8798	− 2.8763
*I (eV)*	6.1093	6.1148
*A (eV)*	2.8798	2.8763
*χ (eV)*	4.4945	4.4955
*η (eV)*	1.6147	1.6192
*S (eV)*^*−1*^	0.3097	0.3088

**Fig. 14:**
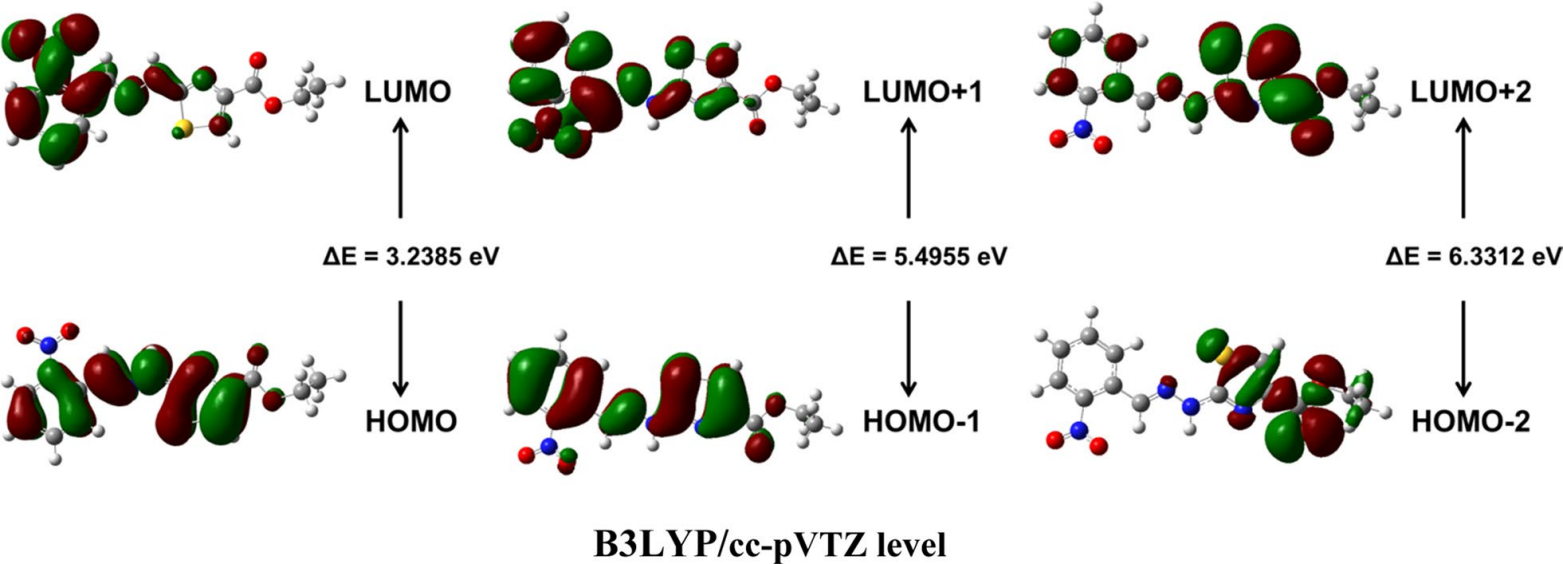
FMOs at B3LYP/cc-pVTZ level

FMO theory assists in the study of photo-electronic properties of the interesting compounds. The energy of HOMO’s and LUMO’s were computed by DFT method using B3LYP level along with 6-311G(d,p) and cc-pVTZ subset levels. The results are presented in Fig. 8 which shows the electronic contribution in HOMO-2, HOMO-1 and HOMO with LUMO, LUMO + 1 and LUMO + 2 of the compound. There is the delocalization of HOMO on all atoms of the molecule except ethyl group while concentration of LUMO at all atoms except ester group (Fig. 14). Theoretical data of HOMO (H) and LUMO (L) energies has been described in Table 4 which shows almost same values of energies for both methods i.e. 6-311G(d,p) and cc-pVTZ [53].

**Table 4:** FMO energies calculated using B3LYP/6–31 + G(d, p) level and B3LYP/cc-pVTZ level

MO(s)	B3LYP/6-311G(d, p)		B3LYP/cc-pVTZ	
	E(eV)	ΔE(eV)	E(eV)	ΔE(eV)
HOMO	− 6.1093	3.2295	− 6.1148	3.2385
LUMO	− 2.8798		− 2.8763	
HOMO − 1	7.6517	5.5062	7.6517	5.4955
LUMO + 1	2.0425		2.0735	
HOMO − 2	7.6471	6.3701	7.6517	6.3312
LUMO + 2	1.2770		1.3205	

### UV–Vis analysis

TDDFT studies (B3LYP method with 6-311G(d,p) and cc-pVTZ basis sets) were carried out in gas phase to get absorption properties. Calculated parameters i.e. wavelength of maximum absorption (*λ*_*max*_), oscillator strength (*f*), excitation energy (*E*^*DFT*)^ and orbitals mainly involved for excitation are listed in Table 5 below [54].

**Table 5:** Wavelength, excitation energy and oscillator strength of compound

Parameters	*λ*_*Exp*_ (nm)	*λ*_*DFT*_ (nm)	*E*^*DFT*^ (eV)	*f*	MO contribution
6-311G(d,p)		436.53	2.8402	0.2548	H → L (70%)
	260,350^a^	340.35	3.6428	0.3228	H → L + 2 (66%)
	256, 338^b^				H-3 → L (19%)
	268, 360^c^	331.35	3.7417	0.0296	H-8 → L (22%) H-3 → L + 1 (44%)
ccPVTZ		437.92	2.8312	0.2341	H → L (70%)
	260,350^a^	342.88	3.6160	0.3467	H → L + 2 (68%)
	256, 338^b^				H-3 → L (14%)
	268, 360^c^	328.85	3.7703	0.0188	H-8 → L (26%) H-3 → L + 1 (47%)
					H-3 → L + 2 (12%) H-1 → L (10%) H → L + 2 (14%)

*λ*_*Exp*_, experimental wavelength; *λ*_*DFT*_, calculated wavelength; *E*, Excitation energy (ev); MO, molecular orbitals; a, THF, b, DMF, c, DMSO

The value of computed absorption maxima (*λ*_*DFT*_) were found to be 436.53, 340.35 and 437.92, 342.88 nm for B3LYP/6-311G(d, p) and B3LYP/ccPVTZ levels respectively with percentage of orbital contribution of [H → L (70%) and H → L + 2 (66%)]. Calculated wavelength (*λ*_*DFT*_) 340.35 and 342.88 employing two different basis sets matched nicely with experimental (*λ*_*Exp*_) value of 350 (DMSO), 338 (DMF) and 360 (THF). It may be concluded that solvent plays its part for the difference between *λ*_*DFT*_ and *λ*_*Exp*_ value (Additional file 1: Figure S1).

### Molecular electrostatic potential (MEP)

**Fig. 15:**
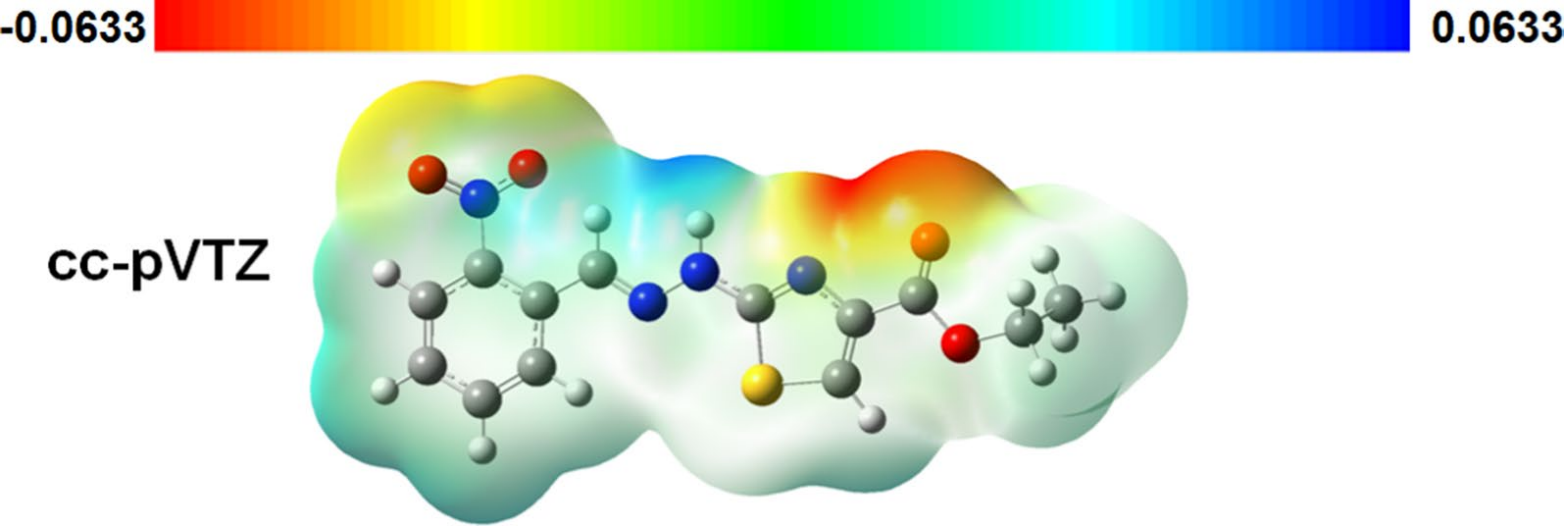
MEP map of compound in gas phase usingB3LYP/cc-pVTZ

The information about electron acceptor and electron-donor regions in a molecule can be easily visualized by MEP. Here the electrostatic potential decreases in the following order red < orange < yellow < green < blue. Molecular electrostatic map of compound is shown in Fig. 15 (B3LYP/cc-pVTZ basis set) where blue and red indicates the areas of strongest attraction and strongest repulsion, respectively. It is concluded that area of strong repulsions i.e. red resides on O1, O2, O3 and N4 atoms while that of strong attractions i.e. blue resides at N2 and N3 atoms, respectively. Therefore, it is assessed that N2 and N3 atom are electron-donor while O1, O2, O3 and N4 atoms are electron-acceptor [55].

### Mulliken population analysis

**Fig. 16:**
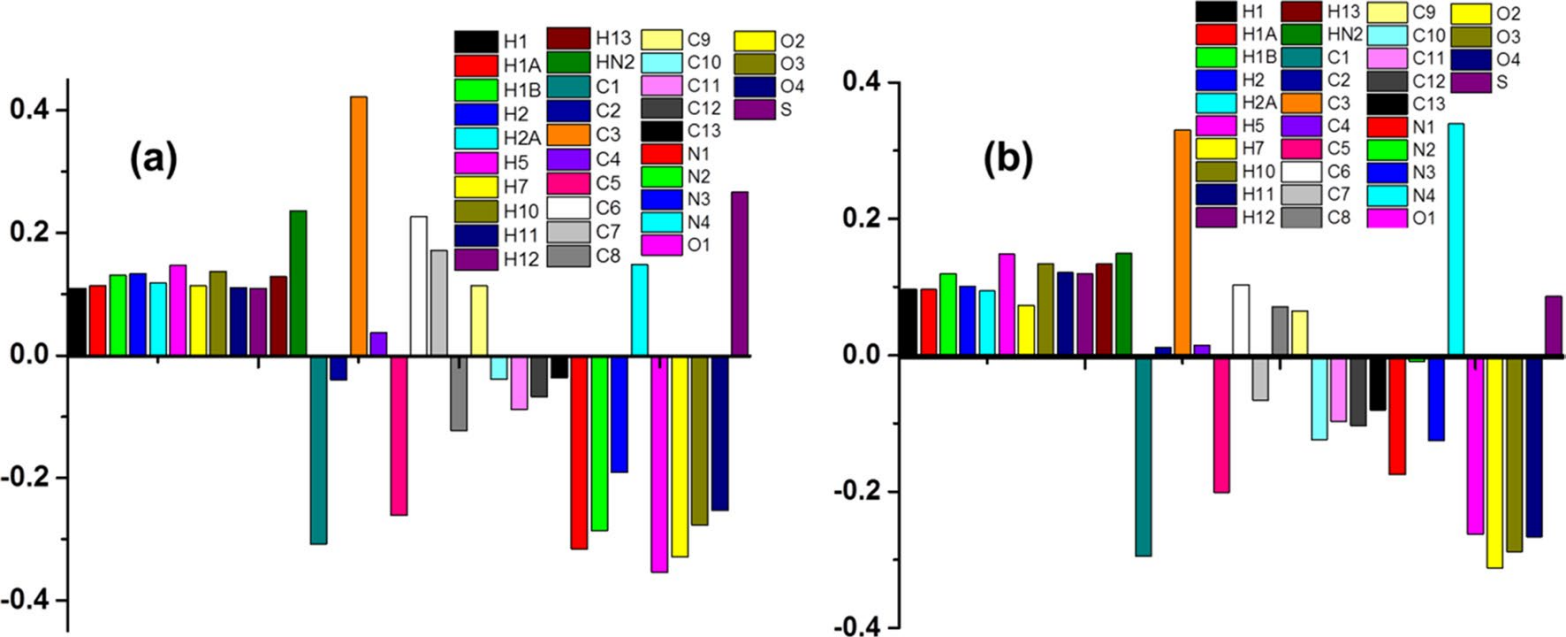
The Mullikan charges diagram of compound (**a**) B3LYP/6-311G(d,p) (**b**) B3LYP/ccPVTZ

The charge distribution on the molecule was calculated theoretically with the help of B3LYP methods using 6-311G(d, p) and cc-pVTZ basis sets (Fig. 16). It is the charge distribution within the molecule, there by, rendering important information about hydrogen bonding within the molecule [54, 55]. It can also be used to characterize the electronic charge distribution in a molecule and the bonding, antibonding, or nonbonding nature of the molecular orbitals for particular pairs of atoms. Mullikan charges of hydrogen atoms are in positive number while negative for carbons except carbon atoms that are attached to oxygen and nitrogen atoms (due to strong electronegativity of O and N atoms). Oxygen and Nitrogen atoms play the vital role in inter- and intra-molecular hydrogen bonding.

### Thermodynamic properties

The vibrational analysis (B3LYP method with 6-311G(d,p) and B3LYP/cc-pVTZ basis sets) was used to calculate the thermodynamical parameters and properties of the compound i.e. entropy (${S}_{m}^{o}$), heat capacity (${C}_{p,m}^{o}$) and enthalpy (${H}_{m}^{o}$). Thermodynamical parameters i.e. entropies, standard heat capacities and enthalpies exhibit a direct relation with temperature. Their correlation equations for both B3LYP/6-311G(d,p) (Eqs. 1–3) and B3LYP/cc-pVTZ (Eq. 4–6) levels have been calculated (Fig. 11) and may be used for further studies of the compound and may serve as guideline for similar compounds. From these correlations it is evident that entropy, enthalpy and heat capacity of the compound (**1)** increases with increase in temperature.

**Fig. 17:**
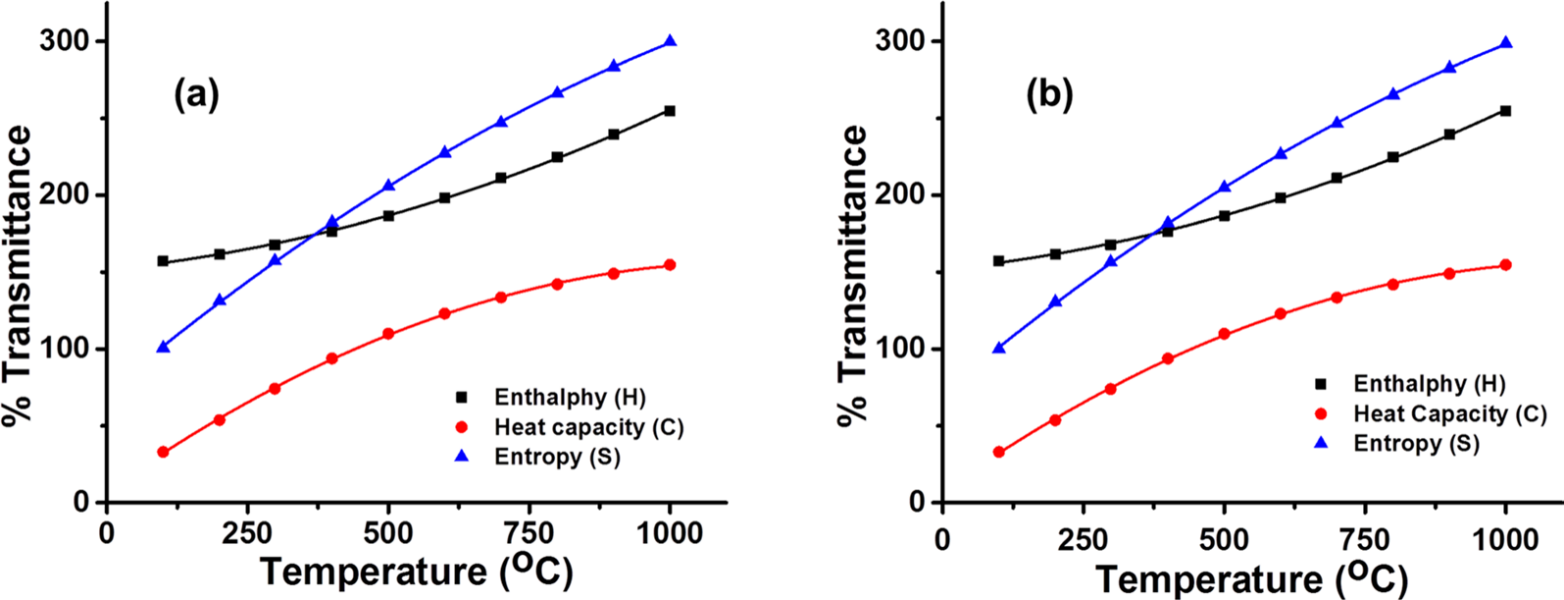
Thermodynamic properties of compound (**a**) B3LYP/6-311G(d,p) (**b**) B3LYP/cc-pVTZ

Heat capacity is due to the rotational and vibrational energy that a molecule possesses. At low temperature there is not enough energy in order to excite many rotations and vibrations, so the slope is small. At very high temperature many energy levels are populated, so the rate of increase becomes constant. The same phenomenon has been observed in case of molecule (**1)** (Fig. 17). As enthalpy is sum of total energies of a molecule, so, it follows the same pattern of slow increase at low temperature but at high temperature it increases steadily due to the dominance of entropy. Entropy of a molecule increases with increase in temperature. The same phenomenon of enthalpy and entropy has also been observed in case of compound (**1)** also.

1 $${S}_{m}^{o}= -8{E}^{-5}{T}^{2}+0.3079T+71.92 \left({R}^{2}=0.9911\right)$$

2 $${H}_{m}^{o}= 7{E}^{-5}{T}^{2}+0.0363T+151.81 \left({R}^{2}=0.9901\right)$$

3 $${C}_{p,m}^{o}=-1{E}^{-4}{T}^{2}+0.2593T+7.5953 \left({R}^{2}=0.9917\right)$$

4 $${S}_{m}^{o}= -8{E}^{-5}{T}^{2}+0.3081T+71.98 \left({R}^{2}=0.9916\right)$$

5 $${H}_{m}^{o}=7{E}^{-5}{T}^{2}+0.0362T+151.92 \left({R}^{2}=0.9924\right)$$

6 $${C}_{p,m}^{o}=-1{E}^{-4}{T}^{2}+0.2588T+7.5854 \left({R}^{2}=0.9913\right)$$

### Comparison of HOMO and LUMO analysis of compound with other derivatives

Careful analysis of molecular orbitals of molecules (**1**–**6)** reveals that except –CO– and OEt groups, HOMO and LUMO are completely delocalized on all atoms. The energy gap (∆E) between HOMO and LUMO is summarized in Table 6 below shows lowest energy gap (3.2385 eV) in case of compound (**1)** having electron withdrawing –NO_2_ substituent while highest energy gap (3.982 eV) in case of molecule (**4)** having methyl substituent. The energy gaps of other molecules are in between (3.819 eV-3.9149 eV) although there is a little difference. This data clearly indicates different substituents (electron donor/electron acceptor) has great effect on electronic properties of the molecule (Table 6). Therefore compound (**1)** possess better capability for electron transfer (lowest ∆E) as compared to other compounds (**2**–**6**) [25].

**Table 6:** Comparison of frontier orbital energies, electronegativity, hardness and softness of compounds (**1–6**)

Parameter/compound	**(1)**	**(2)**	**(3)**	**(4)**	**(5)**	**(6)**
	Present Study	[54]	[25]	[55]	[54]	[55]
***E***_***HOMO***_	− 6.1148	− 5.8849	− 5.4942	− 5.864	− 5.9649	− 5.989
***E***_***LUMO***_	− 2.8763	− 1.9700	− 1.5863	− 1.882	− 2.0568	− 2.170
**∆E**	3.2385	3.9149	3.9079	3.982	3.9081	3.819
***I (eV)***	6.1148	5.8849	5.4942	5.864	5.9649	5.989
***A (eV)***	2.8763	1.9700	1.5863	1.1882	2.0568	2.170
***χ (eV)***	4.4955	3.9274	3.5402	3.873	4.0109	4.0795
***η (eV)***	1.6192	1.9574	1.9539	1.911	1.9541	1.9095
***S (eV)*** ^−***1***^	0.3088	0.2543	0.2559	0.2511	0.2559	0.2618
***Μ***	− 4.4955	− 3.9274	− 3.5402	− 3.873	− 4.0109	− 4.0795

The calculated data of *E*_*HOMO*_ and *E*_*LUMO*_ can be used in order to determine other molecular parameters which are also summarized in Table 6. There is an inverse relationship between energy gap, softness and reactivity of species while a direct relation towards hardness and stability of the molecule. Therefore, compound (**4)** which possess highest energy gap will resist any change indicating low reactivity while compound (**1)** will have high reactivity. The calculated energy gap of compound (**1)** is lowest but ionization potential (6.1148 eV) and electron affinity (2.8763 eV) values of compound (**1)** are greater than others. The hardness of molecule (**1)** is lowest (1.6192 eV) while softness is greatest (0.3088 eV) thus confirming the inverse relationship of energy to softness and direct relationship to hardness, respectively. The calculated values of softness (0.2511 eV–0.2618 eV) and hardness (1.9095 eV–1.9574 eV) of other molecules are comparable (Table 6) [58, 59].

The chemical potential (μ) is related to the stability of a molecule and there is a direct relationship. The value of μ for compound (**1)** is least (− 4.4955 eV) while higher (− 3.5402 eV) for compound (**3)**. The order of chemical potential is as under:

$$\begin{aligned} & \left[ {{\mathbf{1}}\left( {\mu = - {4}.{\text{4955 eV}}} \right)} \right] < \, \left[ {{\mathbf{6}}\left( {\mu = - {4}.0{\text{795 eV}}} \right)} \right] \, < \, \left[ {{\mathbf{5}}\left( {\mu = - {4}.0{1}0{\text{9 eV}}} \right)} \right] \, \\ & \quad < \, \left[ {{\mathbf{2}}\left( {\mu = - {3}.{\text{9274 eV}}} \right)} \right] \, < \, \left[ {{\mathbf{4}}\left( {\mu = - {3}.{\text{873 eV}}} \right)} \right] \, < \, \left[ {{\mathbf{3}}\left( {\mu = - {3}.{54}0{\text{2 eV}}} \right)} \right] \\ \end{aligned}$$

The data also indicates that –CH_3_, –Br and –OH substituent has almost comparable effect whereas –NO_2_ substituent has an immense effect on overall electronic properties of the compound. Based on calculated data of hardness it can be inferred that the compound (**2)** (1.9574 eV) is the most stable and less reactive compared to compound (**6)** (1.6192 eV). Although hardness values of compounds (**2**–**6**) are almost comparable (Table 6).

### Antioxidant assays [57]

Percent free radical scavenging assay (%FRSA), total antioxidant capacity (TAC) and total reducing power (TRP) studies of compound **(1)** exhibit 77.52 ± 0.34 percent scavenging, 144.07 ± 0.16 and 62.13 ± 0.84 µgAAE/mg values, respectively. The %FRSA, TAC and TRP results support the use of compound **(1)** as an antioxidant agent. However, the antifungal and antibacterial studies did not exhibit any significant activity.

## Conclusions

The synthesis of ethyl 2-[2-(2-nitrobenzylidene)hydrazinyl]thiazole-4-carboxylate (**1**) and its characterization by spectroscopic techniques (UV–Vis, FT-IR, ^1^H- and^13^C–NMR), HRMS and SC-XRD is accomplished. The structure was optimized by computational methods. X-rays analysis revealed that molecular configuration is stabilized by C–H…N and C–H…O bonding whereas crystal packing is stabilized by intermolecular N–H…O, C–H…N, C–H…O bonding and weak off-set π–π stacking interaction. Non-covalent interactions that are responsible for crystal packing are further explored by Hirshfeld surface analysis. Moreover, the experimental and theoretical spectroscopic results were in excellent agreement with each other. The MEP map shows that N2 and N3 atom are electron-donor while O1, O2, O3 and N4 atoms are electron-acceptor. The atoms which are electron rich or electron deficient are responsible for intra- and intermolecular attractive or repulsive interaction. The nearly planar geometry is also reflected by the low HOMO–LUMO gape, due to extended conjugation in the molecule. Theoretical data of compounds (**2–6)** has also been compared with that of (**1)** that indicates that –CH_3_, -Br and –OH substituents has almost comparable effect whereas –NO_2_ (Compound **1**) substituent has great effect on overall electronic properties of the compound. The larger value of hardness than softness reflets greater stability of the compound that is important for technological applications. The compound **(1)** was also tested for antioxidant (% FRSA, TAC and TRP studies) and antimicrobial (antibacterial and antifungal) potential. It is concluded based on antioxidant results that compound **(1)** could be used as UV absorber in sunscreen lotions and may protect the skin damage from UV radiations.

## Supplementary Information

**Additional file 1.** Synthesis, crystal structure, Hirshfeld surface investigation and comparative DFT studies of ethyl 2-[2-(2-nitrobenzylidene)hydrazinyl]thiazole-4-carboxylate. **Table S1.** Comparison of the experimental and calculated vibrational wavenumbers (cm^-1^) of compound 1 in gas phase. **Table S2.** Experimental and theoretically calculated ^1^H NMR. **Table S3.** Experimental and theoretically calculated ^13^C NMR.

**Additional file 2.**

## Data Availability

CCDC (Cambridge Crystallographic Data Center) number 1951603 contains the supplementary crystallographic data for the ethyl 2-[2-(2-nitrobenzylidene)hydrazinyl]thiazole-4-carboxylate (**1)**. This data can be obtained free of charge via http://www.ccdc.cam.ac.uk/conts/retrieving.html, or from the Cambridge Crystallographic Data Centre, 12 Union Road, Cambridge CB2 1EZ, UK; fax: (+ 44) 1223-336-033; or e-mail: deposit@ccdc.cam.ac.uk. While the computational data is presented in Additional files 1, 2.

## Abbreviations

CCDC: Cambridge Crystallographic Data Centre
DFT: Density Functional Theory
DMSO-*d*_6_: Dimethyl Sulfoxide-deuterated
FMO: Frontier Molecular Orbital
FRSA: Free Radical Scavenging Assay
FT-IR: Fourier Transform Infrared
HOMO: Highest Occupied Molecular Orbital
HRMS: Hight Resolution Mass Spectrometry
HS: Hirshfeld Surface
IEF-PCM: Integral-Equation-Formalism Polarizable Continuum Model
LUMO: Lowest Unoccupied Molecular Orbital
MEP: Molecular Electrostatic Potential
NMR: Nuclear Magnetic Resonance
ORTEP: Oak Ridge Thermal Ellipsoid Plot
SC-XRD: Single Crystal X-Rays Diffraction
TAC: Total Antioxidant Capacity
TDDFT: Time Dependent Density Functional Theory
TED: Total Energy Distribution
TLC: Thin Layer Chromatography
TOF-ESI: Time of Flight-Electrospray Ionization
TRP: Total Reducing Power
UV–Vis: Ultra violet-visible
