# Supplementary material for: Synthesis, crystal structure, Hirshfeld surface investigation and comparative DFT studies of ethyl 2-[2-(2-nitrobenzylidene)hydrazinyl]thiazole-4-carboxylate
Source: BMC Chem. 2022 Mar 22;16(1):18. doi: 10.1186/s13065-022-00805-1 (PMC8941777; doi:10.1186/s13065-022-00805-1)
Supplement: Supplementary file 1 — Additional file 1. Synthesis, crystal structure, Hirshfeld surface investigation and comparative DFT studies of ethyl 2-[2-(2-nitrobenzylidene)hydrazinyl]thiazole-4-carboxylate. Table S1. Comparison of the experimental and calculated vibrational wavenumbers (cm-1) of compound 1 in gas phase. Table S2. Experimental and theoretically calculated 1H NMR. Table S3. Experimental and theoretically calculated 13C NMR. [file 13065_2022_805_MOESM1_ESM.docx]

**Synthesis, crystal structure, Hirshfeld surface investigation and comparative DFT studies of ethyl 2-[2-(2-nitrobenzylidene)hydrazinyl]thiazole-4-carboxylate**

Muhammad Haroon^1,2^, Tashfeen Akhtar^1*^, Muhammad Yousuf^3*^, Muhammad Nawaz Tahir^4^, Lubna Rasheed^5^, Syeda Saniya Zahra^6^, Ihsan ul Haq^6^, Muhammad Ashfaq^4,7^.

^1^Department of Chemistry, Mirpur University of Science and Technology (MUST), 10250-

Mirpur (AJK) Pakistan.

^2^Department of Chemistry, Government Major Muhammad Afzal Khan (Shaheed), Boys Degree College Afzalpur, Mirpur, (Affiliated with Mirpur University of Science and Technology (MUST), 10250-Mirpur (AJK) Pakistan).

^3^Department of Chemistry, Ulsan National Institute of Science and Technology (UNIST),

Ulsan, South Korea.

^4^Department of Physics, University of Sargodha, Sargodha, Punjab, Pakistan.

^5^Department of Chemistry, Division of Science and Technology, University of Education, Township, Lahore, Pakistan.

^6^Department of Pharmacy, Quaid-i-Azam University, 45320-Islamabad, Pakistan.

^7^Department of Physics, University of Mianwali, Mianwali, Punjab, Pakistan.

email: *^1^[tashfeenchem@must.edu.pk](mailto:tashfeenchem@must.edu.pk); [*^2^nyousuf@postech.](mailto:*2nyousuf@postech.)ac.kr

**Supporting Information**

**Table S1: Comparison of the experimental and calculated vibrational wavenumbers (cm^-1^) of compound 1 in gas phase.**

| **TED** | **Experimental** | **B3LYP/6-311G(d,p)** | | **B3LYP/ccPVTZ** | |
| --- | --- | --- | --- | --- | --- |
|  | **IR** | **ν^β^** | **I_IR_** | **ν^β^** | **I_IR_** |
| ν(H_N2_)96 | 3420 | 3423 | 2.0259 | 3421 | 2.3232 |
| ν(H_C5_)95 | 3270 | 3273 | 0.6768 | 3272 | 0.7469 |
| ν(H_C10-13_)90-94+ ν(H_C7_) 90-94 | 3201 | 3202 | 0.3717 | 3201 | 0.4037 |
| ν(H_C1-2_)85-89 | 3116 | 3111 | 0.828 | 3114 | 1.3453 |
| ν(H_C1-2_)85-89 | 3057 | 3058 | 2.682 | 3055 | 3.4463 |
| ν(H_C1_)85 | 2980 | 2985 | 1.908 | 2982 | 2.2715 |
| ν(C=O_2_)84 | 1685 | 1682 | 26.0631 | 1686 | 31.6107 |
| ν(C_7-13_)79-83+ ν(NO_2_)79-83+ ν(C_7_=N_3_)79-83+ ν(C_6_=N_2_)79-83+ ν(C_4_=N_1_)79-83 | 1571 | 1570 | 1.7811 | 1573 | 2.1494 |
| ν(C_4-5_)77-78+ ν(C_7-13_)77-78 | 1516 | 1520 | 1.0377 | 1518 | 1.1044 |
| ν(H_C1-2_)74-76 | 1477 | 1475 | 0.8865 | 1478 | 0.7469 |
| ν(H_C10-13_)72-73+ ν(H_C7_)72-73+ ν(H_N2_)72-73 | 1438 | 1440 | 2.0115 | 1439 | 2.9216 |
| ν(H_C1-2_)69-71+ ν(H_C7_)69-71 | 1392 | 1390 | 1.3689 | 1393 | 0.9328 |
| ν(NO_2_)66-68+ ν(C_7_=N_3_)66-68+ ν(C_8-13_)+ ν(C_4_=N_3_)66-68 | 1336 | 1332 | 17.1279 | 1334 | 20.6074 |
| ν(H_C2_)65 | 1298 | 1301 | 0.7407 | 1300 | 0.9614 |
| ν(H_C10-13_)62-64+ ν(H_C7_)62-64+ ν(H_N2_)62-64 | 1251 | 1255 | 18.1458 | 1250 | 21.9637 |
| ν(H_C10-13_)61+ ν(H_C7_)61+ ν(H_N2_)61+ ν(C_3_-O_1_)61 | 1244 | 1246 | 32.8086 | 1245 | 44.6853 |
| ν(H_C10-13_)57-60+ ν(H_C7_)57-60+ ν(H_N2_)57-60+ ν(H_C1-C2_)57-60 | 1160 | 1162 | 25.3179 | 1161 | 32.4973 |
| ν(H_C1-2_)55-56+ ν(H_C5_)55-56+ | 1103 | 1105 | 14.0607 | 1104 | 18.1973 |
| ν(C_10-13_)44+ ν(NO_2_)44 | 918 | 916 | 4.698 | 917 | 5.577 |
| ν(H_C10-13_)37-38+ ν(H_C7_)37-38+ ν(H_N2_)37-38 | 848 | 850 | 3.3021 | 849 | 3.3594 |
| ν(C_7-13_)31+ ν(NO_2_)31+ ν(C_7_=N_3_)31 | 742 | 743 | 3.3903 | 742 | 4.1932 |
| ν(H_C7_)26+ ν(H_N2_)26+ ν(H_C1-C2_)26 | 698 | 695 | 4.2381 | 697 | 4.8543 |

IR: Infrared; ν: Wavenumbers scaled with SQM methodology; I_IR_: Infrared intensities (km/mol);

**Table S2: Experimental and theoretically calculated ^1^H NMR**

| **Experimental ^1^H NMR** | **Theoretical ^1^H NMR** | |
| --- | --- | --- |
|  | **6-311G(d,p)** | **ccPVTZ** |
| 8.37 | 8.2656 | 8.58 |
| 8.02 | 8.0054 | 8.24 |
| 7.82 | 7.9941 | 7.75 |
| 7.78 | 7.776 | 7.63 |
| 7.6 | 7.5936 | 7.52 |
| 7.8 | 7.3457 | 7.67 |
| 4.24 | 4.675 | 4.57 |
| 1.28 | 1.6399 | 1.49 |

**Table S3: Experimental and theoretically calculated ^13^C NMR**

| **Experimental ^13^C NMR** | **Theoretical ^13^C NMR** | |
| --- | --- | --- |
|  | **6-311G(d,p)** | **ccPVTZ** |
| 168.2 | 165.9409 | 167.2 |
| 161.36 | 158.1672 | 161.7 |
| 147.94 | 150.8804 | 151.41 |
| 143.32 | 143.7013 | 148.79 |
| 137.62 | 136.1921 | 136.05 |
| 137.44 | 134.5806 | 135.12 |
| 130.4 | 133.1311 | 133.733 |
| 128.71 | 131.3358 | 130.87 |
| 128.13 | 128.5816 | 129.09 |
| 125.16 | 128.4225 | 129.09 |
| 120.17 | 127.5872 | 124.643 |
| 60.93 | 64.6249 | 62.887 |
| 14.63 | 17.6391 | 14.59 |


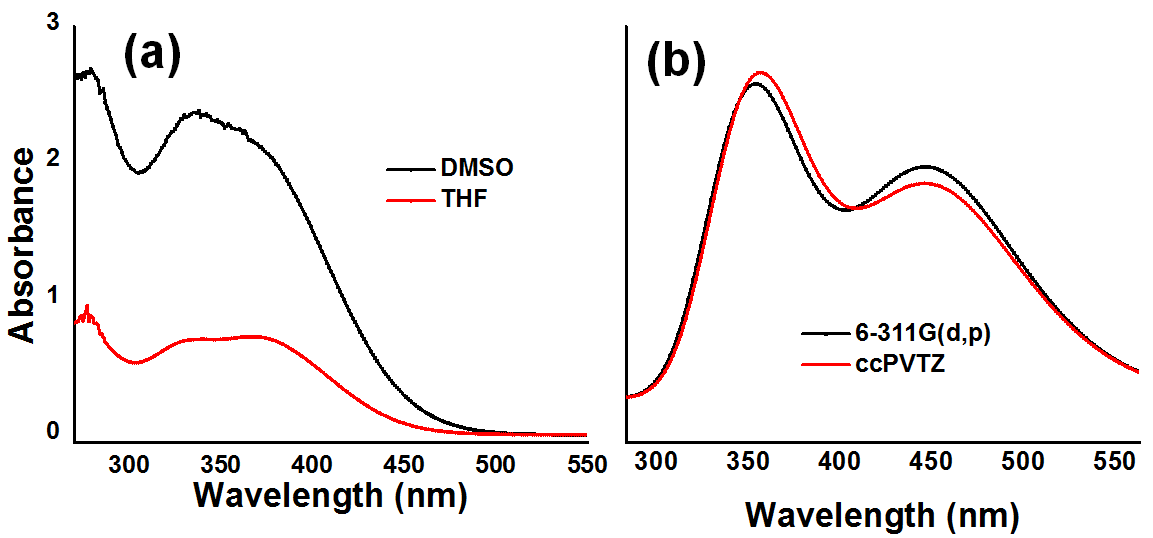


**Figure S1: Experimental and theoretically calculated UV spectra**
